# Perceptual Translucency in 3D Printing Using Surface Texture

**DOI:** 10.3390/jimaging9050105

**Published:** 2023-05-22

**Authors:** Kazuki Nagasawa, Kamui Ono, Wataru Arai, Norimichi Tsumura

**Affiliations:** 1 Graduate School of Science and Engineering, Chiba University, Chiba 263-8522, Japan; 2 Mimaki Engineering Co., Ltd., Nagano 389-0512, Japan

**Keywords:** three-dimensional printing, translucency, perception, texture

## Abstract

We propose a method of reproducing perceptual translucency in three-dimensional printing. In contrast to most conventional methods, which reproduce the physical properties of translucency, we focus on the perceptual aspects of translucency. Humans are known to rely on simple cues to perceive translucency, and we develop a method of reproducing these cues using the gradation of surface textures. Textures are designed to reproduce the intensity distribution of the shading and thus provide a cue for the perception of translucency. In creating textures, we adopt computer graphics to develop an image-based optimization method. We validate the effectiveness of the method through subjective evaluation experiments using three-dimensionally printed objects. The results of the validation suggest that the proposed method using texture may increase perceptual translucency under specific conditions. As a method for translucent 3D printing, our method has the limitation that it depends on the observation conditions; however, it provides knowledge to the field of perception that the human visual system can be cheated by only surface textures.

## 1. Introduction

Translucency is one of the most important appearance properties, along with color and gloss. The term ‘translucency’ has two aspects. The first is the physical aspect, which is characterized by sub-surface light transport. Specifically, it refers to the property that the incidence of light in the sub-surface causes light transport in vertical and horizontal directions through transmission and scattering. Objects with such a property are called translucent objects, and we can clearly observe light scattering by irradiating light on them. The other aspect is the perceptual aspect. This is the visual sensation that humans experience as a result of a complex mixture of many factors, including the physical properties of the object, the geometry of the object, and the lighting environment, all of which reach the human retina. Studies on the human perception of translucency are prolific and were comprehensively reviewed by Gigilashvili et al. [1].

Many materials with translucent appearances exist in the real world, and many attempts have thus been made to reproduce translucent appearances in the fields of computer graphics and three-dimensional (3D) printing. In translucent materials, the light incident in the sub-surface is scattered and light is emitted from a point different from the point of incidence. This phenomenon is called sub-surface scattering and it is described using the bidirectional scattering surface reflectance distribution function (BSSRDF) [2]. The BSSRDF is a model of reflection on a scattering surface with parameters for the object surface position and direction, and it is difficult to handle owing to the large number of parameters. Jensen et al. thus proposed a dipole model that approximates the distribution of light on the surface of an object by giving the internal definition of the BSSRDF in a simpler model [3]. The dipole model first enabled the rendering of translucent materials in the field of computer graphics and is still used widely nowadays. As the model of Jensen et al. assumes homogeneous materials, it is difficult to accurately reproduce translucent objects with a multilayered structure, such as human skin [4]. Donner et al. thus extended the model and proposed a shading model for translucent materials with multilayered structures [5].

Additionally, in the field of 3D printing, research on reproducing a translucent appearance has been conducted in line with the development of printing technology. Early studies reproduced spatially heterogeneous BSSRDFs by combining and stacking multiple translucent materials available for multi-material 3D printers or milling machines at spatially different thicknesses [6,7]. Taking a different approach, Papas et al. proposed a method of controlling sub-surface scattering using a mixture of transparent materials and pigments [8]. In recent years, Takatani et al. proposed a method using ultraviolet (UV) ink [9]. Their method reproduces arbitrary modulation transfer functions (MTFs) by layering an optimized amount of UV ink on top of materials such as wax or eraser rubber.

In contrast to the methods described above, which focus on reproducing the physical properties of translucency, Brunton et al. proposed an approach that focuses on the perceptual aspect of translucency [10]. Considering that the human visual system relies only on simple cues to perceive translucency, they used translucent metamers to reproduce perceptual translucency. They also defined a metric of translucency in line with human perception, a strict definition of which was proposed by Urban et al. [11].

As described above, there have been many studies on translucent reproduction in 3D printing, whereas few works have focused on the perceptual aspect of translucency. We therefore propose a new method for reproducing translucency in 3D printing, focusing on the perceptual aspect of translucency. The idea is to reproduce the cues of human translucency perception using surface textures. Note that the term ‘texture’ in this paper simply means spatially varying color (not micro-scale surface geometry). Although the mechanism of translucency perception in the visual system is not fully understood, it has been suggested that it depends on several cues [1]. Moreover, various cues are commonly related to the surface brightness distribution [12,13]. We thus consider that it is possible to improve perceptual translucency using textures to represent patterns of surface brightness that are effective in translucency perception. In the present study, we considered two main translucent perceptual cues. The first was the high-frequency contrast in non-specular regions shown by Motoyoshi [12], who found that perceived translucency increases with decreasing high-frequency contrast in the non-specular region. The second was the covariance between the surface normal and shading shown by Marlow et al. [13]. In general, the shading intensity covaries with the normal direction of the surface; however, it has been reported that perceptual translucency improves when this covariance is lost.

In this paper, we propose a method of improving perceptual translucency by representing the above two main cues using surface textures. The first step is to create a texture based on the translucent perceptual cues using computer graphics technology. This process includes the calculation of light occlusion patterns and processing of the generated texture images. First, the method of ambient occlusion [14] is adopted to generate a texture that represents shading according to the degree of occlusion due to the object geometry. Next, image processing operations are implemented to represent the translucency perception cues. Finally, the generated textures are applied to the 3D printed object and the effectiveness of the method is validated in subjective evaluation experiments. This paper proposes a method based on the perceptual aspect of translucency; there have been few such methods proposed in the field of translucent reproduction in 3D printing. Furthermore, the study is unique in that it expresses translucency through texture only, even though internal scattering does not actually occur. The motivation for this study is as described below. In the aspect of as a 3D printing method, there are motivations such as reducing the material cost for generating scattering inside the object and the computational cost for optimizing the internal structure; as in conventional methods, by controlling the appearance only with surface textures. Due to the limitation that our method is less effective from a free viewpoint, a use case with a single viewpoint, such as an exhibition in a museum, can be considered. In addition, on the aspect of the perception field, our results of our experiment propose an interesting observation on the suggestion that the human visual system can be cheated by controlling the brightness pattern of an object’s surface.

## 2. Related Works

### 2.1. Color Fabrication

Three-dimensional printing technology has been developed as a method of outputting 3D shapes that have been modeled on a computer or measured using a depth sensor or other means. In addition to the shape output, in recent years, techniques that enable color fabrication have been studied for the purpose of added value. In color fabrication, inkjet 3D printers are widely used, which enables the high-resolution arrangement of various types of UV-cured materials [15]. Considering that UV-curable materials are translucent, Brunton et al. established an error diffusion halftone approach by adjusting the thickness of the white base layer [16]. Moreover, because textures on surfaces are often blurred owing to the scattering properties of the material, approaches have been proposed to optimize the internal structure and enable a more detailed representation [17,18]. Furthermore, this technique has been accelerated using neural networks [19]. As another approach, Babaei et al. proposed contoning, a method of color representation by which inks are stacked [20]. Shi et al. combined halftoning and contoning to reproduce arbitrary spectral reflectance by estimating its layout with a neural network [21]. The 3DUJ-553 inkjet full-color 3D printer, which is used in this study, is available from Mimaki Engineering Co., Ltd. (Nagano, Japan) and it is claimed to be the world’s first inkjet 3D printer capable of representing more than 10 million colors [22].

### 2.2. Fabrication of Translucency

In the field of 3D printing, many studies have investigated the reproduction of translucent appearances. Our study aims to reproduce translucency in 3D printing, and we thus introduce related works in this field. In early works, various scattering behaviors were approximated by overlaying materials with different scattering properties [6,7]. The scattering is calculated using Kubelka–Munk’s multilayer scattering model, which assumes a horizontally semi-infinite media and is therefore not suitable for curved geometries. In addition, the range of BSSRDFs that can be represented is limited to the available materials. Meanwhile, Papas et al. proposed a method of controlling sub-surface scattering by mixing pigments with a transparent base material [8]. Their method realizes data-driven concentration optimization by measuring the scattering properties of mixed materials and creating a database; however, it is limited by the fact that materials are spatially homogeneous and the geometry is created using molds.

Takatani et al. reproduced an arbitrary MTF (modulation transfer function) by layering UV-curable inks on translucent materials such as eraser rubber and wax [9]. They treated the spread of light as a translucency index, which is generally expressed by the point spread function obtained by measuring the intensity distribution of reflected light when a point light source irradiates an object. The MTF is related to the point spread function by the Fourier transform, and the MTF is employed because of its simplicity of measurement. This approach has the limitation that the MTFs that can be produced are not exhaustive, because few varieties of material are used in implementing the method.

There are studies that reproduce arbitrarily the line spread function using multilayered inks with optimized layouts, restricting the modeling object to human skin [23]. Nagasawa et al. proposed a method of reproducing the line spread function as an index of arbitrary skin tone and translucency by representing the epidermal and dermal layers, which are components of human skin, as thin layers of ink and varying the number of each type of layer (i.e., thickness). In this method, an encoder–decoder deep neural network is used to determine the optimal multilayer layout; however, the training data are created using patches actually fabricated by a 3D printer, which is costly. Nagasawa et al. thus developed a method of reproducing multilayer patches on a computer by simulating light scattering using a Monte Carlo method to generate training data [24]. Another approach reproduces the appearance of realistic skin by extracting a concentration map of pigment components from an image of human skin taken with an RGB camera and fabricating a multilayered structure that imitates actual skin [25].

Whereas the above studies focused on defining and reproducing a physical index of translucency, our study focuses on the perceptual aspects of translucency. As an example of a method based on the perceptual aspect of translucency, Brunton et al. proposed an end-to-end pipeline that reproduces color and translucency using the concept of translucent metamers [10]. They focused on the characteristics of human translucency perception and considered that the visual system does not solve inverse problems of light transport, such as estimating physical parameters [1]. To control for perceptual translucency, they used the index A, a perceptually uniform translucency scale. This is the scale proposed by Urban et al. [11] and derived in psychophysical experiments, and a set of isotropic materials are provided that cover this scale. By relating this index A to the A of the RGBA (Red, Green, Blue, A) signal, which is a typical texture format, it is possible to change the translucency linearly in a perceptual manner. Their method changes the horizontal and vertical light transport by editing the voxel arrangement inside the object. Our method differs most from Brunton’s method in that we use only surface textures to represent pseudo translucent perceptual cues and thus do not actually generate scattering phenomena.

### 2.3. Perception of Translucency

Our research is based on the human perception of translucency, and we thus describe related works in the field of perception. Although there is a long history of research on the human perception of translucency, the mechanism is not fully understood. The history and current status of research were summarized in a review by Gigilashvili et al. [1]. In early research, translucency was not treated independently but rather included in the more general issue of visual appearance. Furthermore, in contrast with our present understanding, the visual system was thought to solve the inverse problem of optical processes [26,27]. Later studies led us to consider that the visual system may use empirical, low-level cues and statistics of images [12,28,29,30].

The development of computer graphics has advanced the study of translucency perception. It is necessary to vary these factors systematically when investigating factors that affect perception in the field of psychophysics. The modeling of sub-surface scattering by Jensen et al. [3] made it possible to create experimental stimuli, which accelerated research. Fleming et al. argued that simple image statistics are used in the perception of translucency and discussed various factors that may affect perception [29]. They showed that edge areas contain important cues and back lighting enhances translucency perception. Moreover, they considered that the intensity of shading is the most important cue among the image statistics. Subsequently, Motoyoshi suggested that the luminance contrast of non-specular regions in an image provides a cue for translucency perception and demonstrated this in experiments [12]. The scattering phase function [31] and illumination direction [32] have also been investigated as factors affecting translucency perception. Furthermore, the most informative areas in perceiving translucency have been surveyed [33]. Gkioulekas et al. argued that edges carry important information on sub-surface light transport and proposed representing the physical correlates of translucency by edge profiles [34]. Marlow et al. showed that the lack of covariance between the surface normal and shading information correlates with perceptual translucency [13]. On this basis, they created an image in which the shading is not covariant with the surface shape and demonstrated that it is possible to create the illusion of translucency even though the object is opaque. While most studies have used computer-generated images, psychophysical experiments using real objects have become possible with the development of technology that allows 3D modeling with arbitrarily changed sub-surface scattering properties [10,35]. The observation that humans frequently move the object and their head in perceiving the translucency of a real object [36] suggests that it is important to use real objects in psychophysical studies. In a recent study by Kiyokawa et al. [37], experiments were conducted to consider the possibility that perceived translucency depends on the 3D shape inferred from the surface gloss. The results suggest that perceived translucency can be explained by incongruence between the 3D shapes used to generate the specular and non-specular components of the image.

## 3. Making Textures for Perceptual Translucency

The purpose of this study is to generate perceptual translucency by applying a surface texture that reproduces the cues of translucency perception. To achieve this, we design textures based on two types of cues. The first cue for translucency perception is the high-frequency contrast as proposed by Motoyoshi [12]. When humans observe an object, a phenomenon is observed whereby the perceived translucency increases as the contrast, especially at high frequencies in the non-specular region, decreases. This phenomenon is considered to be due to sub-surface scattering, a physical property of translucent objects. The light that reaches areas that would normally be in shadow reduces the contrast. In addition, this phenomenon more likely occurs in areas with detailed structure (having high spatial frequency) because thinness allows light to penetrate easily. On the above basis, we consider that it is possible to represent translucency by pseudo brightening areas with a high degree of occlusion and reproducing a reduction in contrast.

The second cue for translucency perception is the covariance between the surface normal and shading as proposed by Marlow et al. [13]. In general, a surface is brighter when its normal faces the light source, and the farther the normal is from the light source direction, the stronger the shading on the surface. It is observed that humans perceive translucency when the covariance between the normal direction and shading is lost for some reason. We therefore consider that translucency can be represented by losing covariance between the normal direction and shading.

Both of the above cues are related to the shading intensity distribution (i.e., brightness distribution) of the object’s surface, and we consider that it is possible to reproduce the cues using textures.

### 3.1. Ambient Occlusion

As described previously, the objective of this study is to create textures that change the distribution of the apparent shade intensity. Shading results from the occlusion of light by the geometry. In addition, shading is greatly affected by changes in the lighting environment. Considering these two factors, a base texture is created using the method of ambient occlusion. This method calculates the intensity of shading according to the occlusion of ambient light by the geometry [14]. The target is ambient light, which is basically present in any lighting environment, and the system is thus highly robust against changes in the environment.

In computer graphics, light reaching each point on an object is divided into direct light, which is emitted directly from a light source, and ambient light, which reaches the object after being diffused by the surrounding environment. In this case, the ambient light component is often approximated by a constant because the number of rays involved in ambient light is enormous and the computational cost is high. However, the number of rays of ambient light reaching each point actually depends on how occluded the point is by the surrounding environment. Accordingly, the method of ambient occlusion [14] calculates the degree of occlusion at each point and multiplies it by the ambient light component (a constant) to determine the effect of ambient light. Ambient light components approximated using this can be texturized. We refer to such textures as ambient occlusion textures in this paper.

**Figure 1:**
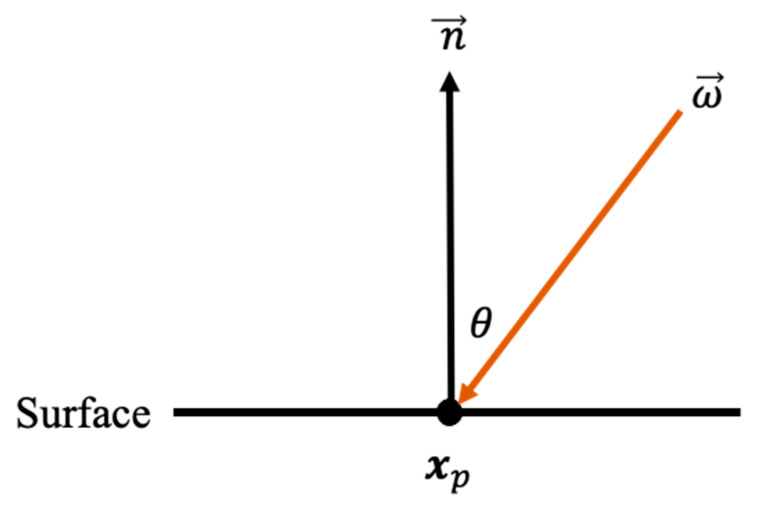
Overview of the ambient occlusion term, indicating whether the ray from the direction $\vec{\omega }$ to the point $x_{p}$ is occluded.

The degree of occlusion is expressed as the ambient occlusion term $A_{p}\;$in the situation shown in Figure 1 and Equation (1):

(1) $$A_{p}=\frac{1}{\pi }\int_{\Omega }^{\;}V\left(x_{p},\vec{\omega }\right)\cos\theta \;d\omega =\frac{1}{\pi }\int_{0}^{2\pi }\int_{0}^{\frac{\pi }{2}}V\left(x_{p},\vec{\omega }\right)\cos\theta \sin\theta \;d\theta d\phi ,$$

where the function V is a binary function that describes whether the point p at position x is occluded from the direction $\vec{\omega }$. Integrating this function V over the hemisphere gives the degree of occlusion.

**Figure 2:**
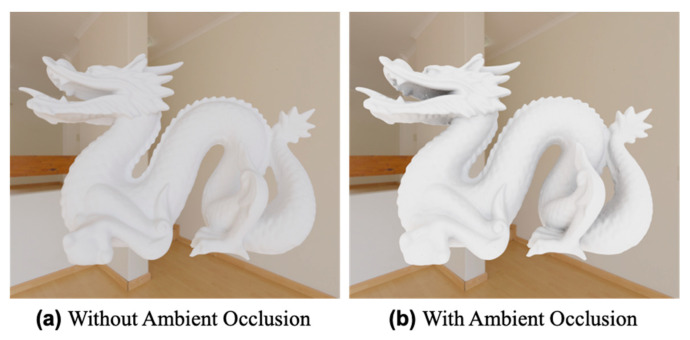
Natural shading added by applying the method of ambient occlusion.

**Figure 3:**
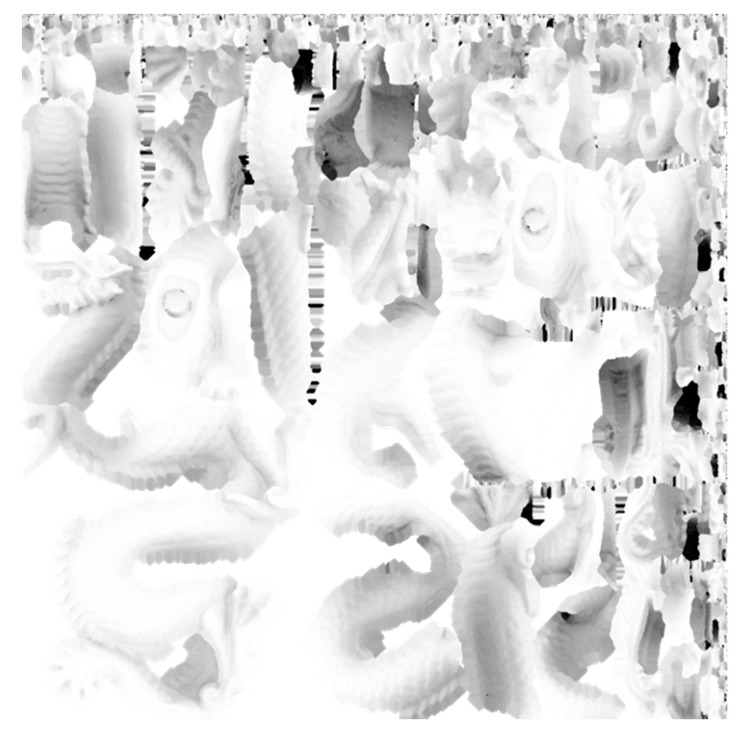
Image of a surface texture obtained using the method of ambient occlusion, unfolded onto a plane.

In this study, we use Blender [38], which is a free modeling and rendering software with the method of ambient occlusion built in [39]. The results of applying the method of ambient occlusion are shown in Figure 2. The geometry of a dragon is imported from the Stanford 3D Scanning Repository [40]. It is seen that shading is added according to the degree of occlusion. The ambient occlusion result in Figure 2b is exported as a texture image, resulting in the image shown in Figure 3 unfolded on a plane.

### 3.2. Texture Processing

Adopting the above method, we create a texture that reproduces translucent perceptual cues by manipulating the ambient occlusion texture. There are three specific processes: gray-level inversion, gray-level range limitation, and gamma correction. To implement all three operations in a single operation, Equation (2) is applied to all pixel values I (before processing) in the texture image:

(2) $$I^{\prime }=\left(256-threshold\right)\times {\left(\frac{255-I}{255}\right)}^{\frac{1}{\gamma }}+threshold$$

where $I^{\prime }$ is the pixel value after processing, threshold is the threshold of the gray-level value to be used, and γ is the gamma value in gamma correction. The effect of each operation is adjusted using threshold and γ as parameters.

We next describe the objectives of each operation. We first explain gray-level inversion. The luminance of the ambient occlusion texture is set so that the areas where ambient light is occluded are darker and the areas where it is not occluded are brighter. In contrast, our method aims to reduce or invert contrast by brightening the shaded area to make it appear as if there is sub-surface light transport. We therefore invert the gradations of the ambient occlusion texture to produce a texture that is lighter in the occluded areas and darker in the non-occluded areas. However, the large difference in brightness between the occluded and non-occluded areas may not be appropriate for reducing contrast. We therefore implement a gray-level range limitation.

In an ambient occlusion texture having an inverted gray level, the non-occluded areas are dark, and we thus restrict the gray-level range of the texture image to the high-luminance range. For example, if threshold is set at 128, all pixels have pixel values within the range from 128 to 255; if threshold is set at 1, the range will be 1 to 255. The gray-level range is adjusted according to the cues of translucency perception that are to be reproduced.

**Figure 4:**
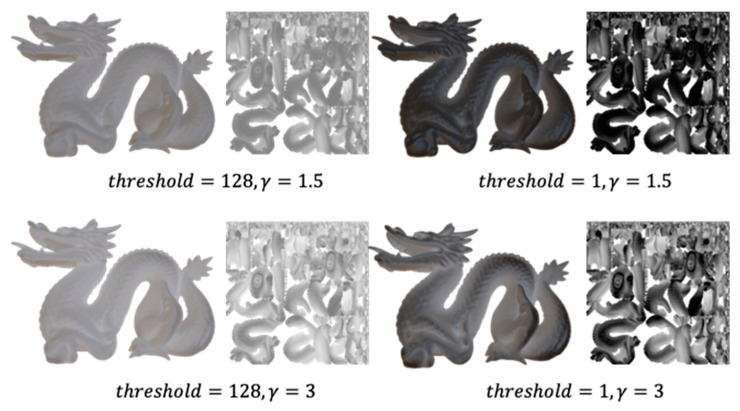
Examples of textures with different parameter values. threshold is the threshold of the gray-level value to be used (1–255) and γ is the gamma value used in gamma correction.

We finally explain the purpose of gamma correction. It has been shown that thin or detailed regions of a structure are important in translucency perception [32]. Therefore, it is necessary to have a large effect even in areas where the degree of occlusion is relatively small (i.e., thin and detailed areas). To achieve this, a gamma correction is applied to expand the effect of reducing shadows to areas with smaller occlusion. Figure 4 shows examples of textures created by varying the parameters threshold and γ. A texture image is shown on the right side of each panel and a rendered image with the texture applied is shown on the left side. It is seen that the parameters can be adjusted to generate textures with different characteristics.

### 3.3. Setting Parameters

Our method reproduces the two translucent perceptual cues mentioned previously. Accordingly, we define indexes with which to evaluate the applicability to each cue and determine the parameters threshold and γ. To enable image-based parameter optimization, we propose a method using rendered images of textured objects.

First, multiple combinations of the parameters threshold and γ are selected to generate a set of textures. The threshold of the gray-level value to be used threshold is set at 1, 64, 128, and 192. The gamma value γ is set at several values between 0.5 and 3, which are known to be empirically valid, namely 0.5, 0.7, 1.0, 1.5, 2.0, 2.5, and 3.0. These parameters are combined to create 28 different rendered images. The number of parameter combinations is limited considering the cost of rendering. Rendering is performed using Blender [38]. For the lighting environment, Lebombo, a high-dynamic-range environment map of a room with sunlight, was obtained from Poly Haven [41] and used to represent a typical diffuse lighting environment. Examples of rendered images are shown to the left of each item in Figure 4. All examples of rendered images with 28 combinations of two parameters are provided in Figure S1 in the Supplementary Materials.

The appropriate parameters are selected by using the 28 images which were rendered using the method described above. We first discuss the first objective, contrast reduction. This objective is based on the observation that light penetrating the occluded area weakens the shading and the resulting reduction in contrast improves perceptual translucency [12]. The texture of the proposed method is designed to brighten the occluded areas and weaken the shading, and we thus select the parameter to maximize this effect. For calculating the contrast, we use the Michelson contrast expressed by:

(3) $$C=\frac{I_{max}-I_{min}}{I_{max}+I_{min}},$$

where $I_{max}$ is the maximum pixel value and $I_{min}$ is the minimum pixel value in the image. In this study, we experimentally used Michelson contrast, which is easy to calculate. This calculation is performed on a rendered image of a 3D object to which a texture has been applied (images such as the left side of Figure 4). The calculation method follows the literature [12] by calculating the contrast in multiple spatial frequency sub-bands and averaging the results. Sub-band images are generated by applying a Gaussian band-pass filter with a bandwidth of 20 in the frequency space. Nine sub-band images are created, and the contrast is calculated for a total of 10 images including the original image. Here, a mask is applied to extract only the object region from the rendered image. In this process, the sub-band image has a high-contrast boundary between the object and background owing to the effect of the band-pass filter, and the mask is therefore blurred to eliminate this effect. This masking process also eliminates the effects of extremely dark/light pixel values occurring between the background and the object. Finally, the contrast is calculated for the object regions in the 10 obtained images, and the parameter with the smallest average contrast is determined as the optimal parameter. In this paper, the texture, whose parameters are determined by this index, is referred to as texture 1.

**Figure 5:**
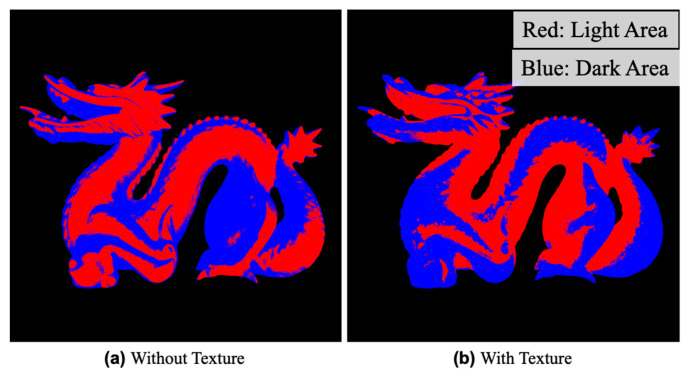
Overview of encoding shading patterns. The light and dark areas, which are divided by the median of luminance on the surface, are respectively shown in red and blue. (**a**) is an example of without texture and (**b**) is an example of with texture. It is seen that the pattern differs greatly depending on the presence or absence of texture.

We next discuss the second objective, limiting covariance between the surface normal and shading. This objective is based on the observation that translucency is perceived when the covariance between the direction of the surface normal and shading is lost [13]. The normal direction and shading intensity generally covary in the opaque case (without texture). Therefore, adjusting the texture so that the distribution of the shading intensity is different from that in the opaque case would improve perceptual translucency. Accordingly, the difference in the intensity pattern of the shading from the opaque case is used as an indicator. The calculation method of this indicator is described below. First, considering the encoding of the distribution of shading on the surface, we define light and dark areas. In the case of an opaque object, the non-occluded area is bright, and the occluded area is dark; we thus consider these as light and dark areas, respectively. In a rendered image, the median pixel value in the object region of the image is used as a boundary with which to divide pixels into light and dark areas. The pattern of shading encoded by the above process is shown in Figure 5. The index is calculated by comparing the number of pixels with different signs relative to the rendered image without texture (Figure 5a). The parameters having the largest value for this index are selected as being optimal. In this paper, the texture, whose parameters are determined by this index, is referred to as texture 2.

## 4. Experiment

This section describes the validation of our method for representing perceptual translucency with textures. Textures created using the method described in the previous section were applied to an object for fabrication using a 3D printer. We validated the effectiveness of our method by conducting a subjective evaluation experiment on the perceptual translucency of the fabricated samples.

### 4.1. Fabrication of Samples

**Figure 6:**
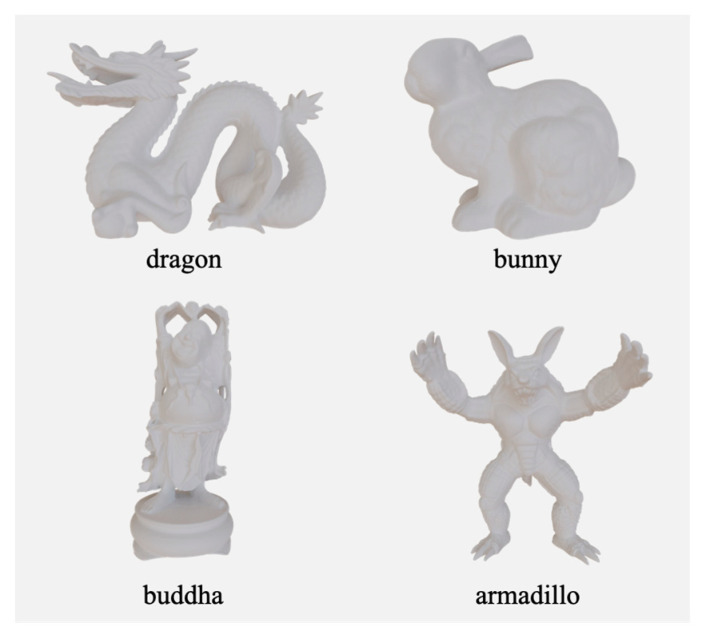
Geometries used in the experiment [40]: a dragon, bunny, Buddha, and armadillo.

**Figure 7:**
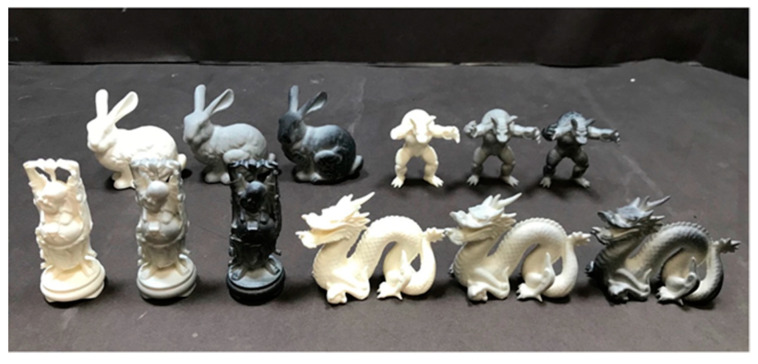
Fabricated samples. For each geometry, from left to right, the order is without texture, with texture 1, and with texture 2.

We used four geometries, namely, a dragon, bunny, Buddha, and armadillo as shown in Figure 6, taken from the Stanford 3D Scanning Repository [40]. For each geometry, we created a texture whose parameters were determined using the two indexes described in the previous section. In addition to these eight samples, non-textured samples were fabricated to validate the effect of the texture. The bulk was fabricated entirely with 100% white ink and was physically opaque. The 3D printer used in this experiment was a Mimaki Engineering 3DUJ-553 [22]. Figure 7 shows the fabricated samples. Close-up photos of each object are provided in Figure S2 in the Supplementary Materials.

### 4.2. Experimental Design

#### 4.2.1. Scale of Translucency

In validating the effectiveness of the proposed method, a subjective evaluation experiment was conducted to evaluate the translucency of each sample. In preparing for the experiment, we determined the definition and scale of translucency, the lighting environment, and the observation environment. The following is a description of each item.

**Figure 8:**
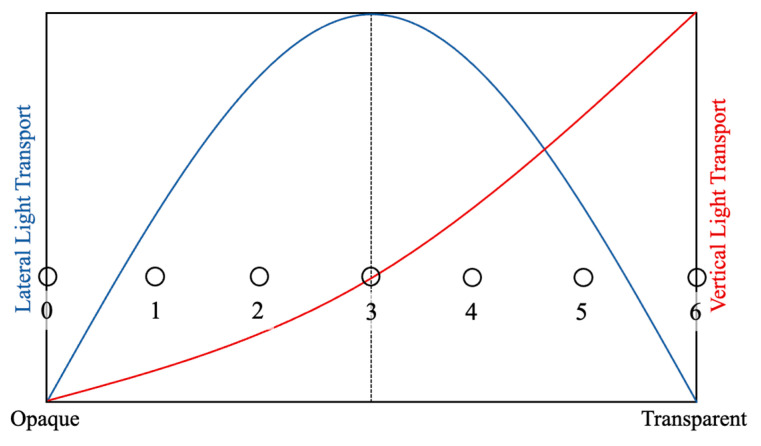
Concept of the translucency scale in the experiment. The scale has seven levels, as indicated by the circles (Referenced from [10]).

**Figure 9:**
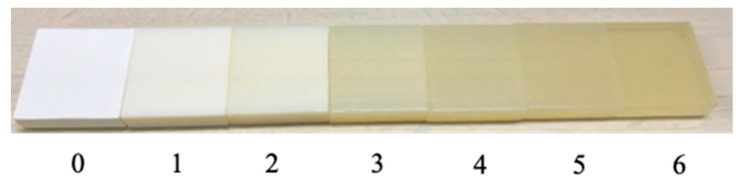
Samples corresponding to our scale. The density of the scatterer decreases from 0 to 6, approaching transparent.

We first discuss issues in considering definitions and scales of translucency. In the field of translucency perception, the term translucency is regarded as having no clear definition [1]. Commonly used psychophysical scaling methods, such as the adoption of magnitude estimation and rank order, are not appropriate in this case because of this ambiguity in the definition of translucency. Instead, many studies adopt matching tasks involving computer graphics [29,32]. Although it would have been preferable to conduct a matching task in the present experiment, it is difficult to adjust parameters in real time in an experimental setting using real objects, making it impossible to perform the task. Therefore, in this experiment, we established our own definition and scale of translucency and held a session to explain them to participants before the evaluation task. We referred to the relationship between scattering and transparency described in the literature [10] and defined translucency as an index determined by the relationship between horizontal and vertical light transport. When an object changes from opaque to transparent, it is known that the horizontal and vertical light transport changes as shown in Figure 8. The horizontal light transport (i.e., spread of light due to sub-surface scattering) does not change monotonically, and it is thus combined with the vertical (transmission) to uniquely define the degree of translucency as shown in Figure 8. To enable subjects to recognize differences in appearance due to changes in the degree of scattering, samples with varying scatterer densities were created using a 3D printer and presented to the subjects. Figure 9 shows samples with scatterer density varying from opaque (light does not penetrate into the interior at all) to transparent (light penetrates into the interior but is not scattered at all), and we assign values on our scale. Considering the balance between task difficulty and granularity, we designed a seven-level evaluation task as indicated by the circles in the figure.

#### 4.2.2. Lighting Environment

**Figure 10:**
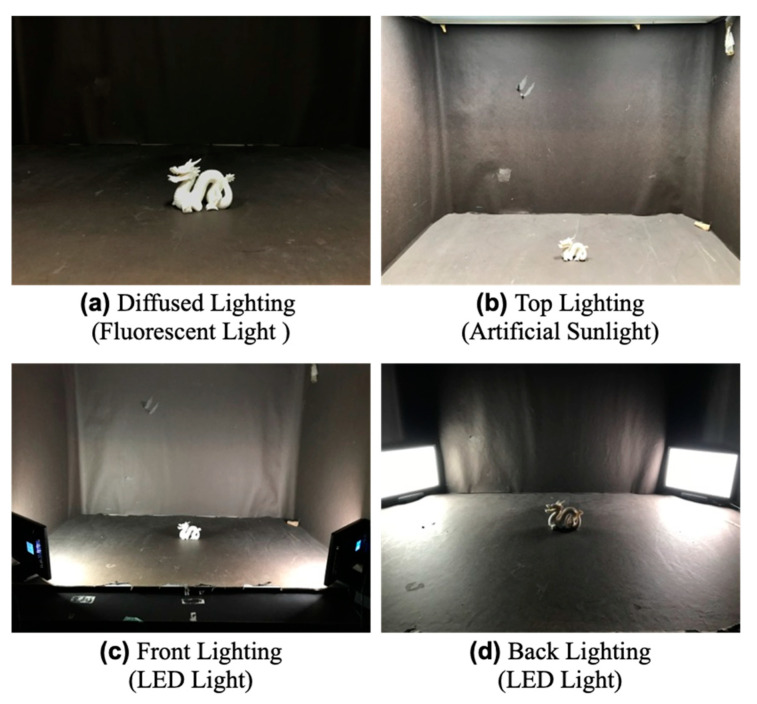
Four lighting environments in the experiment.

The following is a description of the lighting environment. In the present experiment, four lighting environments were prepared as shown in Figure 10. We prepared multiple environments because it is known that the lighting direction affects the perception of translucency [1]. The first environment was of fluorescent light. The method of ambient occlusion is expected to be highly effective under such diffused illumination because it takes ambient light into account. The second environment was of artificial sunlight. Such lighting is commonly used in psychophysical experiments, and the booth used in the present experiment had top lighting. The third and fourth environments were of front and back lighting with illumination from light-emitting diodes. On the basis that back lighting enhances translucent perceptual cues more than front lighting in studies of translucency perception [29,32], we used front- and back-lighting environments.

#### 4.2.3. Observation Conditions

**Figure 11:**
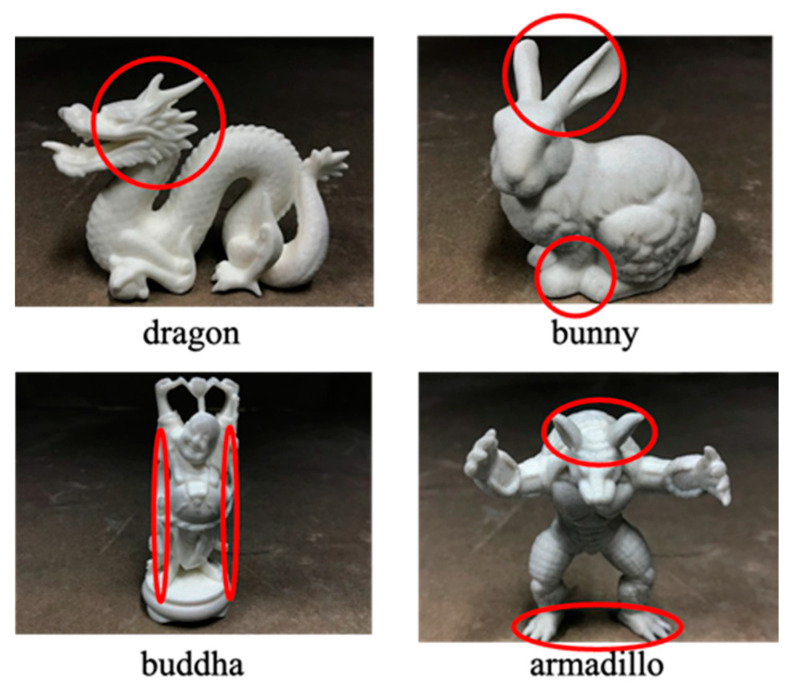
Local observation areas of the different objects.

We finally describe the observation conditions. We conducted the experiment by observing the whole object and by observing a local area of the object. This was conducted knowing that important information as cues for translucency perception is enriched on edges, in thin areas, and in detailed shapes [32]. The areas of local observation for each geometry are indicated by red circles in Figure 11. All the local areas had thin or detailed structure. In local area observations, subjects were shown the entire object and then asked to focus on a specified point.

#### 4.2.4. Experimental Setup

Experiments were performed under a total of eight conditions combining the lighting environments and observation conditions. The 12 samples described in the previous section were targets of observation. The objects were placed in a lighting booth at an observation distance of 60 cm. There were 10 participants, specifically 9 males and 1 female aged in their twenties.

In addition to the above experiment, we conducted an experiment under the condition that the participant could move the object freely. This experiment was based on reports that people move objects and their head when observing translucent objects [36]. This experiment was conducted for only one type of lighting environment, namely, the fluorescent lighting environment, and the whole and local observation conditions.

### 4.3. Results

Table 1 gives the average evaluation values across participants for each experimental condition. Conditions with increased scores compared to no texture are indicated by red values. Under most conditions, at least one of the textures has the effect of increasing perceptual translucency. This indicates that the representation of translucency by texture may be effective; however, the representation lacks robustness against the observation environment. The change in appearance for different scores can be referred to in Figure 9. Note, however, that this is for reference only, as human perception cannot be quantified.

Table 2 compares the results for the observation areas. Each evaluation value is the average for each lighting environment and texture type. For geometries other than the bunny, it is seen that the observation of local areas with thin and detailed structures has higher perceptual translucency than the observation of the whole. We consider that the reason for the higher evaluation value for the observation of the whole bunny is that the thinness and complexity of the local area are lower in the case of the bunny.

Table 3 compares the results for lighting environments. Each evaluation value is the average of the observation areas and texture types. It is seen that the lighting environment providing the highest perceptual translucency is different for each geometry. The effectiveness of our method is thus affected greatly by the interaction between the geometry and lighting environment.

Table 4 compares the results for the fixed view and dynamic view. In this comparison, the lighting environment is fixed as being under fluorescent light. The results show that moving the object reduces the perceptual translucency for most geometries. This is thought to be due to the fact that the interaction with the illumination is changed by adding the movement condition, and the areas that were produced as pseudo shadows are perceived to be texture. In contrast, we consider that the dragon has many detailed shapes, which makes it difficult to recognize the texture.

### 4.4. Discussion

**Figure 12:**
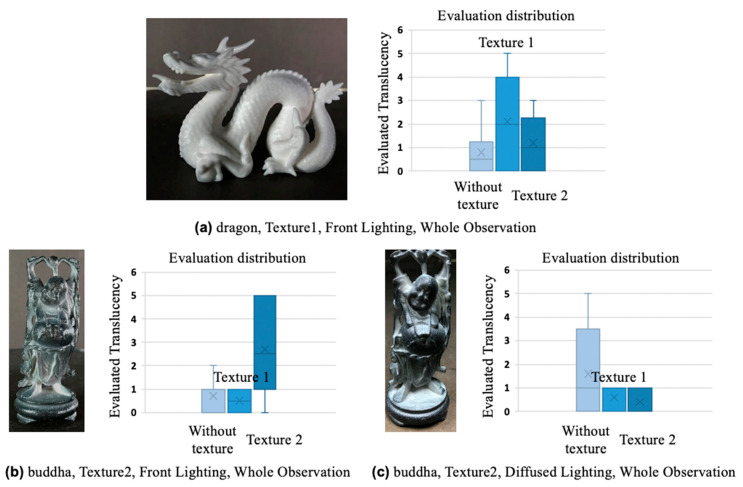
Examples of conditions with particularly high/low evaluation values of translucency (**a**); (**b**) examples of a high evaluation value, and (**c**) an example of a low evaluation value. The graph to the right of each example shows the distribution of evaluations. “x” at box plots represents the mean value.

We discuss the conditions for which there were particularly high and low evaluations of translucency. Figure 12a shows an image of the dragon object with texture 1 taken under the condition of front lighting. Note that this image was captured by a camera and therefore differs from the image perceived by human vision, and the graph to the right of each example shows the distribution of evaluations. Under this lighting condition, the evaluation value is particularly high in the whole-object observation. We consider that the effect of contrast reduction by texture 1 was appreciable under this lighting condition. Some of the participants who perceived high translucency also perceived a wet appearance. The possibility of a correlation between wetness and translucency is suggested and will be the subject of future research. Figure 12b shows an image of the Buddha object with texture 2 taken under the condition of front lighting. Under this lighting condition, the evaluation value is particularly high in the whole-object observation. This is because the extreme brightening by texture 2 is concentrated in areas with detailed structure. There are thus many cues for translucency perception under this condition. Finally, Figure 12c shows an image of the Buddha object with texture 2 taken under the condition of diffused lighting. Under this lighting condition, the texture reduces the perceptual translucency in the whole-object observation. Most of the participants commented that the object appeared to have black paint on it, and it is thus assumed that this lighting condition caused the texture to be perceived as paint rather than as shadows.

Our method assumes that texture creation targeting ambient light is robust against changes in lighting conditions. However, the results were found to depend strongly on the object geometry and lighting. Comparison of appearance in the rendering environment and in the four lighting environments used in the experiment is provided in Figure S3 in the Supplementary Materials. Note that the appearance of photos is different from what is actually perceived by a human because it was only captured by a camera.

## 5. Limitations

This study was designed with the idea that ambient occlusion (assuming uniform illumination) could be used to create textures that are independent of the lighting environment; however, we confirmed that the results are dependent on the lighting environment, viewpoint, and geometry. Therefore, it is difficult for this method to improve perceptual translucency in a generic way at this time.

In addition, as can be seen from the results in Table 4, the effectiveness of our method decreases with the free (dynamic) viewpoint. We consider that this is due to the fact that the textures are created using images rendered from a single viewpoint.

## 6. Conclusions and Future Works

We proposed a method of representing perceptual translucency in 3D printing using textures. We focused on the cues by which humans perceive translucency and enhanced perceptual translucency by reproducing the cues through surface textures. The textures were designed to reproduce translucent perceptual cues relating to the distribution of the shading intensity. We proposed an image-based method using computer graphics for optimizing the parameters used in texture creation. Subjective evaluation experiments were conducted to validate the effectiveness of this method, and the results showed that the method had a certain effect in improving perceptual translucency. However, this method is still difficult to use as a general-purpose translucent 3D printing method because it is highly dependent on the lighting environment, viewpoint, and geometry. Therefore, as future work, we consider that it is necessary to analyze what kind of textures are effective under certain conditions and to create textures suitable for those conditions. Furthermore, we plan to verify the effectiveness of the combination of the two methods proposed in this study.

For the proposed method in this paper, it is necessary to consider using a measure that uses information from all pixels, such as RMS contrast, for the contrast calculation on texture creation. Moreover, our scale in this experiment was used only for the lower half of the values, because for large values of the scale, the appearance is closer to transparent than to translucent (Figure 9 can serve as a reference for the relationship between each value on the scale and appearance). Therefore, our scale of translucency has room for improvement and should be considered as future work.

In addition, we believe that this study contributes to knowledge in the field of perception by suggesting the point at which the human visual system can be cheated only by changes in the brightness of an object’s surface, even though no sub-surface scattering actually occurs.

Although the present study focused on reproducing perceptual translucency, we suggest the possibility of expressing a unique appearance that changes depending on the angle of view and lighting environment by combining our texture with conventional methods of altering scattering characteristics. Furthermore, we consider implementing the method of obtaining shading patterns from rendering results of objects with sub-surface scattering characteristics as future work. Gigilashvili et al. claim that this method has the effect of altering perceptual translucency [42].

## Figures and Tables

**Table 1:** Mean evaluation values of the participants. Conditions with increased scores compared to no texture are indicated by red values.

Lighting Environment	Diffused	Diffused	Top	Top	Front	Front	Back	Back
Observation Area	Whole	Local	Whole	Local	Whole	Local	Whole	Local
dragon(Without Texture)	0.6	1.4	1.6	0.3	0.8	1.9	2	1.6
dragon(Texture1)	0.5	1.6	2.4	1.6	2.1	2.1	0.4	1.3
dragon(Texture2)	0.7	0.7	1	0.6	1.2	0.7	1.5	1.3
bunny(Without Texture)	0.8	1.2	2	0.7	1.1	1.9	1.9	1.5
bunny(Texture1)	1.5	1.2	2.3	2	2	0.9	1.1	1.7
bunny(Texture2)	1.5	1.8	0.4	1.9	0.7	1.6	2	0.6
buddha(Without Texture)	1.6	1.6	0.6	1.6	0.7	1.4	2.1	0.4
buddha(Texture1)	0.6	2.3	1.4	0.6	0.5	1.8	2.5	1.6
buddha(Texture2)	0.4	1.8	2.5	1.8	2.7	2.3	0.7	2.1
armadillo(Without Texture)	1.2	1.5	0.9	1.4	0.9	1.3	1.3	0.6
armadillo(Texture1)	2.5	2.3	0.4	1.8	0.5	2.3	1.6	0.7
armadillo(Texture2)	1.1	2	2.6	1.4	2.3	2.3	0.3	1.7

**Table 2:** Comparison of evaluation values for observation areas. Red values are high values per geometry.

	Whole	Local
dragon	1.23	1.26
bunny	1.44	1.42
buddha	1.36	1.61
armadillo	1.30	1.61

**Table 3:** Comparison of evaluation values for lighting environments. Red values are high values per geometry.

	Diffused	Top	Front	Back
dragon	0.92	1.25	1.47	1.35
bunny	1.33	1.55	1.37	1.47
buddha	1.38	1.42	1.57	1.57
armadillo	1.77	1.42	1.60	1.03

**Table 4:** Comparison of evaluation values between the fixed view and dynamic view. Red values are high values per geometry.

	Fixed View	Dynamic View
dragon	0.92	1.23
bunny	1.33	1.13
buddha	1.38	1.37
armadillo	1.77	1.25

## Data Availability

The data presented in this study are available in the article and supplementary.

## Supplementary Materials

The following supporting information can be downloaded at: https://www.mdpi.com/article/10.3390/jimaging9050105/s1, Figure S1: All 28 combinations of two parameters used in this study and rendered image with texture applied. *Threshold* is the threshold of the gray level value to be used (1–255) and γ is the gamma value used in gamma correction; Figure S2: All of fabricated samples with diffused lighting (fluorescent light); Figure S3: Comparison of appearance in the rendering environment and in the four lighting environments used in the experiment.
