# Supplementary material for: Perceptual Translucency in 3D Printing Using Surface Texture
Source: J Imaging. 2023 May 22;9(5):105. doi: 10.3390/jimaging9050105 (PMC10219096; doi:10.3390/jimaging9050105)
Supplement: Supplementary file 1 [file jimaging-09-00105-s001.zip › jimaging-2379938-supplementary.pdf]

Supplementary material

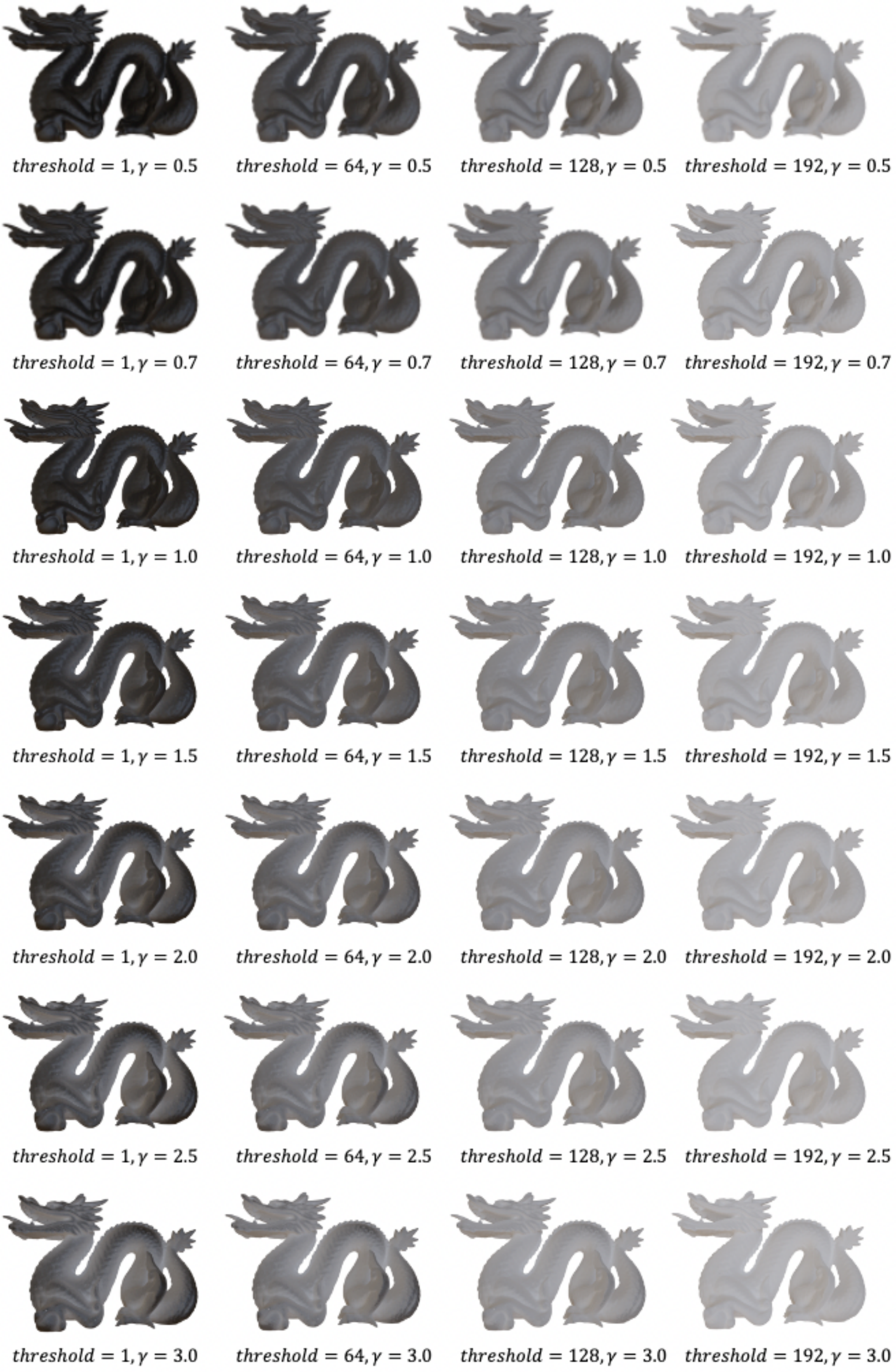

Figure S1. All 28 combinations of two parameters used in this study and rendered image with texture applied. $threshold$ is the threshold of the gray level value to be used (1-255) and γ is the gamma value used in gamma correction.

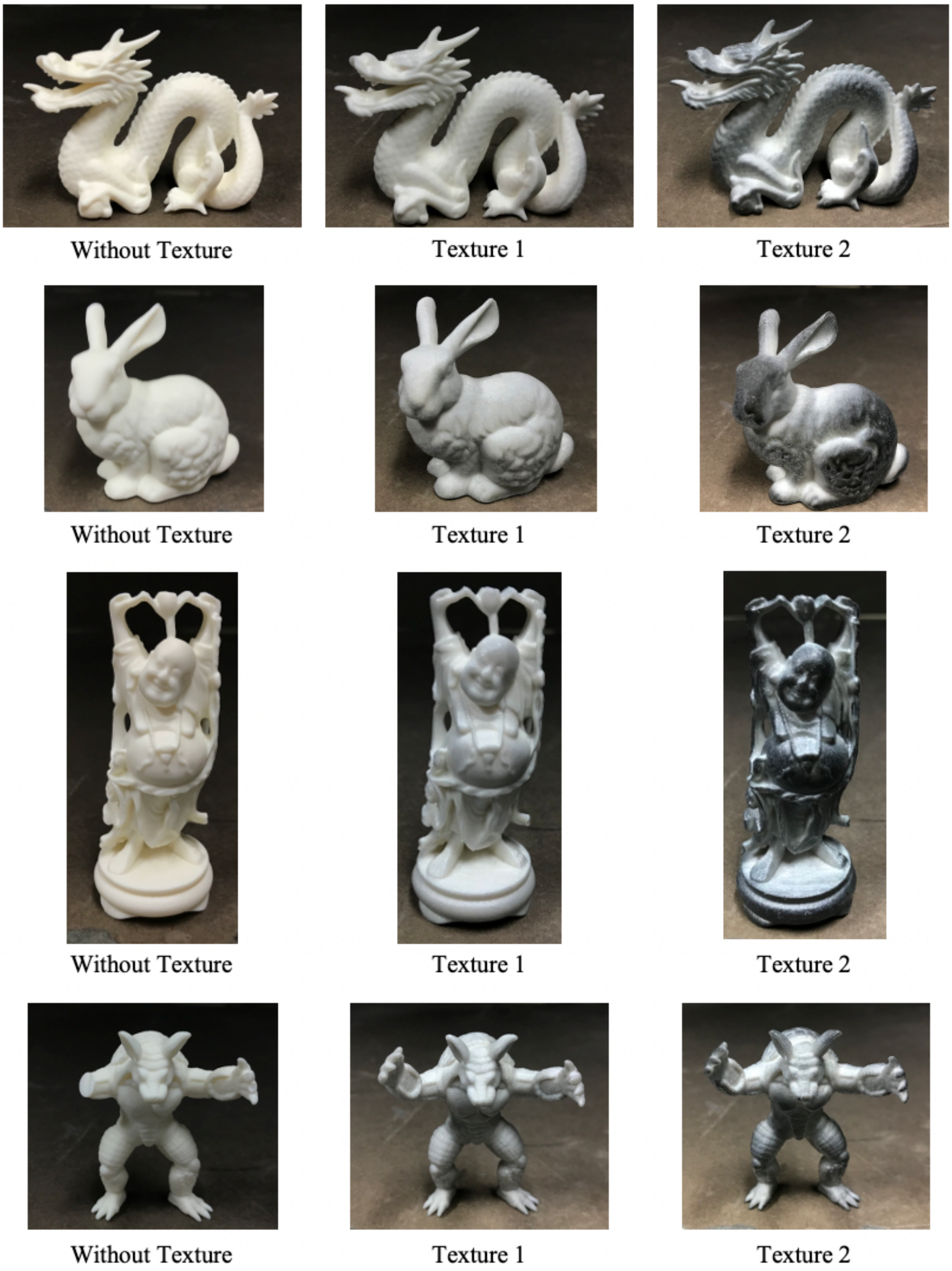

Figure S2. All of fabricated samples with diffused lighting (fluorescent light).

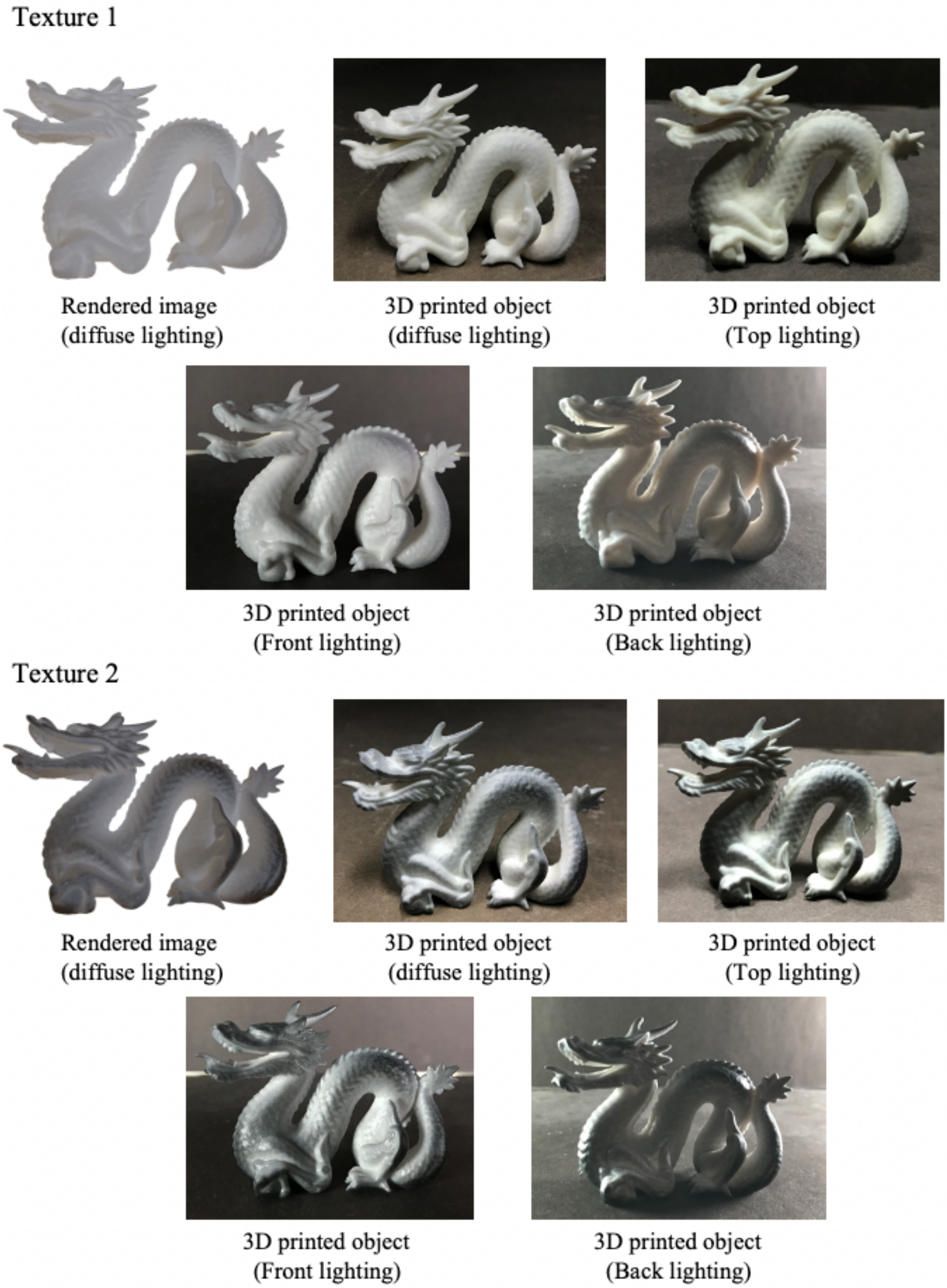

Figure S3. Comparison of appearance in the rendering environment and in the four lighting environments used in the experiment.
